# Supplementary material for: Caregivers’ use of herbal and conventional medicine to treat children with sickle cell disease at Jinja Regional Referral Hospital, Eastern Uganda: A cross-sectional study
Source: PLoS One. 2023 Sep 8;18(9):e0291008. doi: 10.1371/journal.pone.0291008 (PMC10490855; doi:10.1371/journal.pone.0291008)
Supplement: Attachment — Submitted filename: Response to Reviewers.doc [file pone.0291008.s002.doc]

**RESPONSES TO COMMENTS**

**REVIEWER ONE**

The manuscript describes a phenomenon that is common in Africa. Some findings were quite interesting. The use of herbal remedies among individuals with tertiary education, over 50% of the cohort used herbal remedies. The demographics were also quite interesting. Most of the respondents were unmarried. It would have been important to distinguish the caregivers that were actual parents in the study population.

**RESPONSE: We thank the reviewer for these nice compliments and observations.** **The suggestion to distinguish caregivers who were actual parents from those who were not is very important as this would show whether there were differentials in terms of herbal/conventional drug administration by the actual parents and those who were not. However, in our study questionnaire, we did not include a question that would distinguish the actual caregiver who was a parent from one who was not and therefore, we are not able to carry out that categorization. We acknowledge this as a limitation and have clearly stated it in the discussion section on page 27.**

**REVIEWER TWO**

The manuscript is well written, the results are sound, and support the conclusions made. The methodology is largely logical except for a few things.

1. The sampling criteria/procedure is not elucidated. Did you consecutively or randomly sample participants? Please clearly include it in the text.

**RESPONSE: We thank the reviewer for the pleasant observations and comments. Study participants were recruited by consecutive sampling. The sampling criteria have been clearly indicated under the sampling strategy within the materials and methods section. For details, please see pages 6 and 7.**

2. Did you pretest the questionnaire you developed from the literature for suitability in your setting? Please provide more information on this.

**RESPONSE: Yes, Questionnaires were pretested on 5 caregivers of children with sickle cell disease who were randomly chosen from the sickle cell clinic. More information has been provided under the data collection tools within the methods section. For details, please see page 7.**

3. Usually sickle cell disease manifests from around 6 months, by recruiting caretakers of patients from 1 year old, did you intentionally exclude those infants receiving treatment? If so, clearly state it with reason.

**RESPONSE:** **We intentionally excluded children with sickle cell disease of 1 year and below because, at 6 months, when the disease has just started to manifest, most caregivers do not have conclusive findings of the diagnosis of the disease and are most likely to rely on conventional medicine for disease management. However, with time, caregivers are more likely to look out for other remedies with the realization that the disease is chronic, incurable, and burdensome. We have, however, acknowledged this as a limitation in the discussion section. For details, see page 27.**

**Additional changes**

1. We have edited the paper to improve grammatical expression and clarity of statements

2. We have improved the formatting of the paper in light of the authors' guidelines

3. We have edited the references to align them with the journal's referencing style.
