# Supplementary material for: Caregivers’ use of herbal and conventional medicine to treat children with sickle cell disease at Jinja Regional Referral Hospital, Eastern Uganda: A cross-sectional study
Source: PLoS One. 2023 Sep 8;18(9):e0291008. doi: 10.1371/journal.pone.0291008 (PMC10490855; doi:10.1371/journal.pone.0291008)
Supplement: Attachment — Submitted filename: Response to Reviewers.docx [file pone.0291008.s003.docx]

**RESPONSES TO COMMENTS**

**EDITOR’S COMMENTS**

1. You should define what you mean by `Care givers` in the context of your study.

**RESPONSE: We thank the reviewer for the keen comments and observations.**

**The definition of caregivers in the context of this study has been defined in the Methods and materials section under study population on page 6.**

2. Tables 4 and 5 should reflect the confidence intervals beyond presenting p-values

**RESPONSE: For both tables 4 and 5, analysis was re-done and the confidence intervals have been reflected. This can be seen in the Results section on pages 16 for Table 4 and page 17 for Table 5.**

3. Regarding the results in Table 6, what statistical tests were done with this particular data? There are no significant levels presented and no confidence intervals.

**RESPONSE: We thank you for the inquiry.**

**A binary logistic regression was re-run to compute the p-values and the confidence intervals. This has been reflected in Table 6, in the Results section on page 19. The statistical tests done have also been re-emphasized on page 18.**

4. The theory of planned behavior should be explained clearly in your introduction.

**RESPONSE: We thank you for the observation.**

**The theory of planned behavior has been clearly explained in the Introduction section on page 4.**

5. The discussion should show the strengths of the paper and implications of clinical management of children with sickle cell in relation to the study findings

**RESPONSE: Thank you for the observations.**

**Some of the strengths of the study were already highlighted in the Discussion section, nevertheless; we have added another strength of the paper. This is clearly indicated in the Discussion section on page 27. The implications of clinical management of children with sickle cell in relation to the study’s findings have also been added to the Discussion section on page 27.**

6. Adult age in Uganda begins at 18 years. How then can you say you included children 1-18 years?

**RESPONSE: Thank you for the inquiry.**

**The study only included children from 1 year to those below 18 years. This has been re-emphasized in the method and materials sections under the sampling procedures and study population. This has been clarified on pages 6 and 7.**

**Other changes made in the manuscript**

For Table 3, we opted to make some changes as the table does not show associations but rather a comparison of the proportions of caregivers who used a combined therapy against those who did not use based on the selected sickle cell disease management characteristics**.**

But for the rest of the tables, Tables 4, 5, and 6, the confidence intervals have been reflected beyond the p-values, as suggested by the editor.
